# A Natural Glucan from Black Bean Inhibits Cancer Cell Proliferation via PI3K-Akt and MAPK Pathway

**DOI:** 10.3390/molecules28041971

**Published:** 2023-02-19

**Authors:** Peng Li, Yihua Hu, Lingmin Zhan, Jiaqi He, Jingwu Lu, Chunyan Gao, Weijun Du, Aiqin Yue, Jinzhong Zhao, Wuxia Zhang

**Affiliations:** 1 Department of Basic Sciences, Shanxi Agricultural University, Taigu 030801, China; 2 College of Agriculture, Shanxi Agricultural University, Taigu 030801, China

**Keywords:** black bean, polysaccharide, anti-cancer activity, cell cycle arrest, PI3K/Akt, MAPK

## Abstract

A natural α-1,6-glucan named BBWPW was identified from black beans. Cell viability assay showed that BBWPW inhibited the proliferation of different cancer cells, especially HeLa cells. Flow cytometry analysis indicated that BBWPW suppressed the HeLa cell cycle in the G2/M phase. Consistently, RT-PCR experiments displayed that BBWPW significantly impacts the expression of four marker genes related to the G2/M phase, including *p21*, *CDK1*, *Cyclin B1*, and *Survivin*. To explore the molecular mechanism of BBWPW to induce cell cycle arrest, a transcriptome-based target inference approach was utilized to predict the potential upstream pathways of BBWPW and it was found that the PI3K-Akt and MAPK signal pathways had the potential to mediate the effects of BBWPW on the cell cycle. Further experimental tests confirmed that BBWPW increased the expression of *BAD* and *AKT* and decreased the expression of *mTOR* and *MKK3.* These results suggested that BBWPW could regulate the PI3K-Akt and MAPK pathways to induce cell cycle arrest and ultimately inhibit the proliferation of HeLa cells, providing the potential of the black bean glucan to be a natural anticancer drug.

## 1. Introduction

Anticancer drugs usually possess both strong cytotoxicity and side effects [1,2,3,4]. Thus, it is extremely urgent to develop low-toxicity and non-toxic drugs for cancer treatment. In recent years, numerous studies have indicated the potential anticancer effects of natural products, especially those plants with the homology of food and medicine, which play a significant role in developing new anticancer drugs due to their unique advantages of low toxicity and wide sources [5]. The black bean, as one of the common medicinal and edible homologous plants, contains abundant anthocyanin, saponins, carbohydrates, proteins, vitamins, and trace elements [6]. These ingredients endow black beans with various abilities, including anticancer and immunoregulation [7,8,9]. However, till now, only some soy-derived small molecular compounds, such as protease inhibitors, saponins, and isoflavones were found to have anti-cancer effects. The macromolecular components, such as polysaccharides, whose immunoregulatory activities have been fully examined, lack studies about their anti-cancer activities. Based on these facts, we expected to find anti-cancer polysaccharides from black beans.

In the present study, a polysaccharide BBWPW was purified from black beans. Its structure was characterized by chemical composition analysis, molecular weight detection, monosaccharide composition, infrared spectroscopy, and nuclear magnetic resonance analysis. Furthermore, we examined its anti-cancer activity and explored the potential molecular mechanisms.

## 2. Results

### 2.1. Purification and Component Identification of Black Bean Polysaccharide

#### 2.1.1. Extraction and Purification of the Polysaccharide from Black Bean

The crude polysaccharide of black bean was extracted by hot water and the yield was 17.20%. After fractionation on the DEAE-52 fiber column, further purification and elution were performed on the SephadexG-100 gel column. A pure component with a yield of 8.65% was obtained and named BBWPW (Supplementary Materials Figure S1).

#### 2.1.2. Molecular Weight Determination

The molecular weight of a polysaccharide is closely related to its biological activities [10]. It was eluted as the asymmetric peak on HPGPC at 41.95 min (Supplementary Materials Figure S2), indicating that BBWPW is a relatively pure component, and the average molecular weight of BBWPW was 2.4 × 10^4^ Da.

#### 2.1.3. Monosaccharide Composition of BBWPW

**Figure 1:**
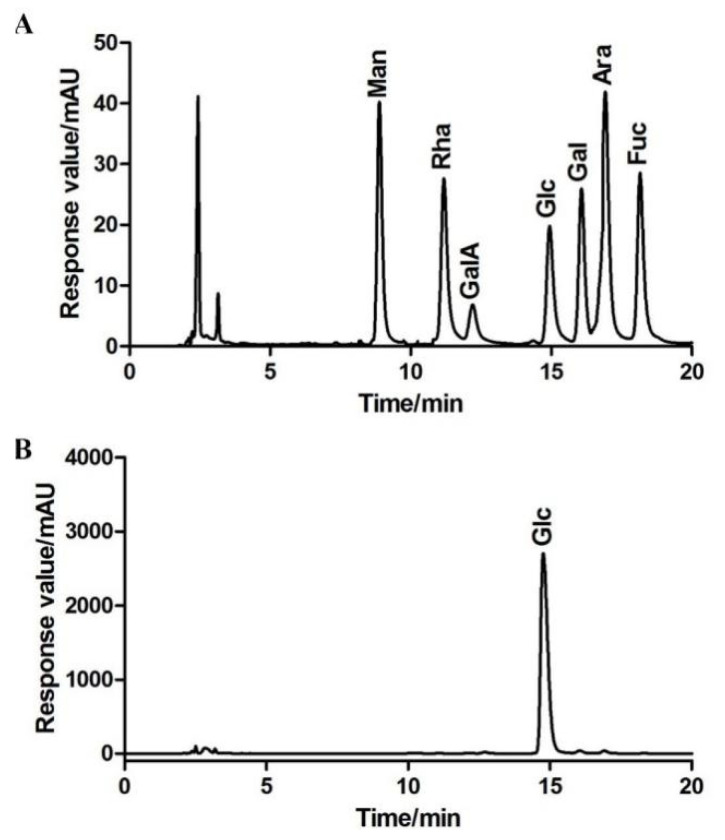
Determination of monosaccharide standards (**A**) and BBWPW (**B**).

The colorimetric analysis showed that BBWPW contained 98.88 ± 0.59% carbohydrate. This indicated that BBWPW is a neutral polysaccharide. According to the peak time of the standard monosaccharides, HPLC analysis results showed that BBWPW is mainly composed of glucose (Figure 1).

### 2.2. Structural Characterization of Black Bean Polysaccharide

#### 2.2.1. The Surface Morphology of BBWPW

SEM was used to observe the surface morphology of particles in biological materials and samples [11]. The SEM of BBWPW revealed that the surface of BBWPW showed a flaky and gully structure with random cracks (Supplementary Materials Figure S3A,B). We speculated that BBWPW may have a certain viscosity and a large intermolecular force [12].

#### 2.2.2. Infrared Spectra Analysis

BBWPW was analyzed among 400–4000 cm^−1^ by IR analysis (Supplementary Materials Figure S3C). The absorption peak near 3305.82 cm^−1^ and 1400.93 cm^−1^ were caused by the stretching vibration of -OH and the variable angular vibration of C-H, respectively. The absorption peaks between 950 and 1200 cm^−1^ were attributed to the stretching vibrations of C-O-C and C-O [13].

#### 2.2.3. NMR Analysis

We utilized NMR spectroscopy to detect the structural information of BBWPW. The C/H chemical shifts were assigned and listed in Table 1 according to the monosaccharide composition result and 2D NMR spectra.

**Figure 2:**
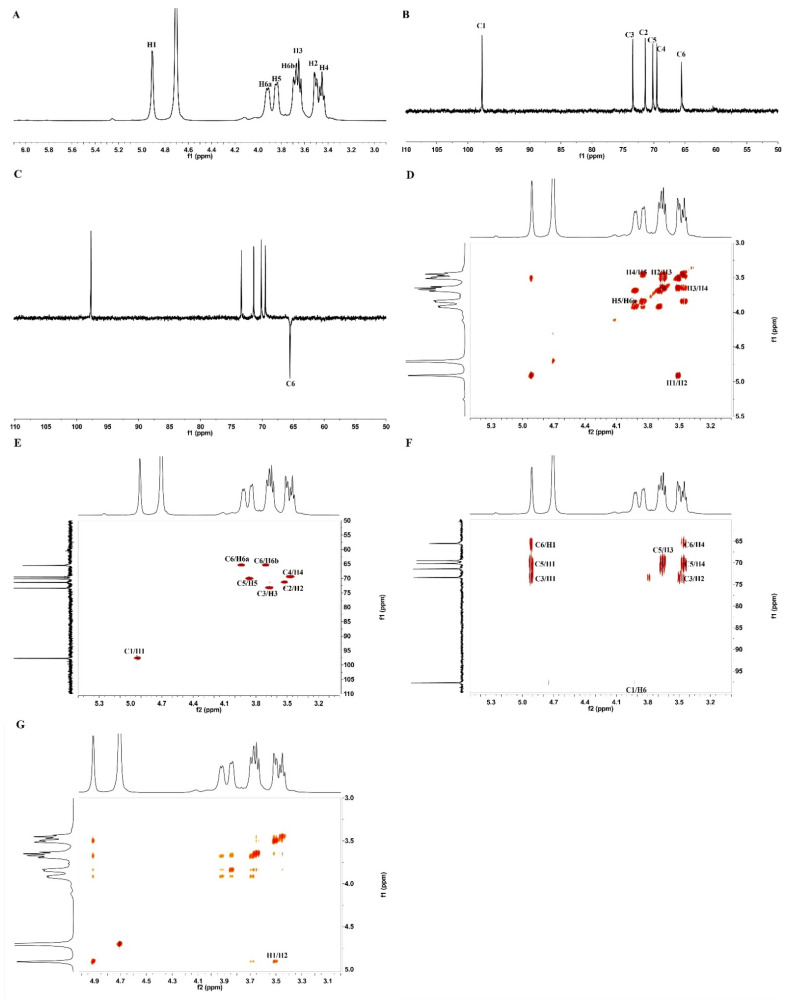
NMR spectra of BBWPW. (**A**) ^1^H NMR, (**B**) ^13^C HMR, (**C**) DEPT 135, (**D**) ^1^H-^1^H COSY, (**E**) HSQC NMR, (**F**) HMBC and (**G**) NOESY spectra of BBWPW.

Based on the above structural composition analysis, BBWPW was composed of glucose (Glc). Thus, in the ^1^H NMR spectrum of BBWPW (Figure 2A), the signal at 4.91 ppm was attributed to the anomeric hydrogen atoms H1 of Glc. As shown in Figure 2B, the anomeric carbon atoms C1 of Glc at δ 97.77 ppm were less than 100 ppm, which suggested that the Glc residue was α-linked. In the DEPT-135 spectrum, the inverted signal at δ 65.60 ppm was attributed to C-6 of α-Glc (Figure 2C). 65.60 ppm migrate to the lower field, indicating substitution at C-6 of α-Glc.

The COSY (Figure 2D) and HSQC (Figure 2E) spectrums were used to assignate the other signals of BBWPW in 1D NMR. The C1 and H1 signals of α-1,6-Glc were δ 97.77 and 4.91 ppm in the HSQC. The cross peaks at δH/H 4.91/3.52, 3.52/3.65, 3.65/3.45, 3.45/3.85, and 3.85/3.93 ppm were detected in ^1^H–^1^H COSY, which suggested that the signals at δ 4.91, 3.52, 3.65, 3.45, 3.85, and 3.93 ppm corresponded to H1, H2, H3, H4, H5, and H6a of the residue α-1,6-Glc, respectively. According to HSQC, C1–C6 of residue α-1,6-Glc were δ 97.77, 71.41, 73.40, 69.57, 70.22, and 65.60 ppm, respectively.

The glycosyl residues, backbone, and substitution sites of BBWPW are confirmed by the HMBC spectrum (Figure 2F). The cross peak at δ 97.77/3.93 ppm represented the correlation between C-1 and H-6a of residue α-1,6-Glc. Combining the data of monosaccharide composition, BBWPW should be an α-1,6-glucans. The cross-peaks of H1 and H2 in the NOESY spectrum (Figure 2G) also indicate that the Glc residue was alpha configuration.

### 2.3. BBWPW Inhibits the Proliferation of Cancer Cells

**Figure 3:**
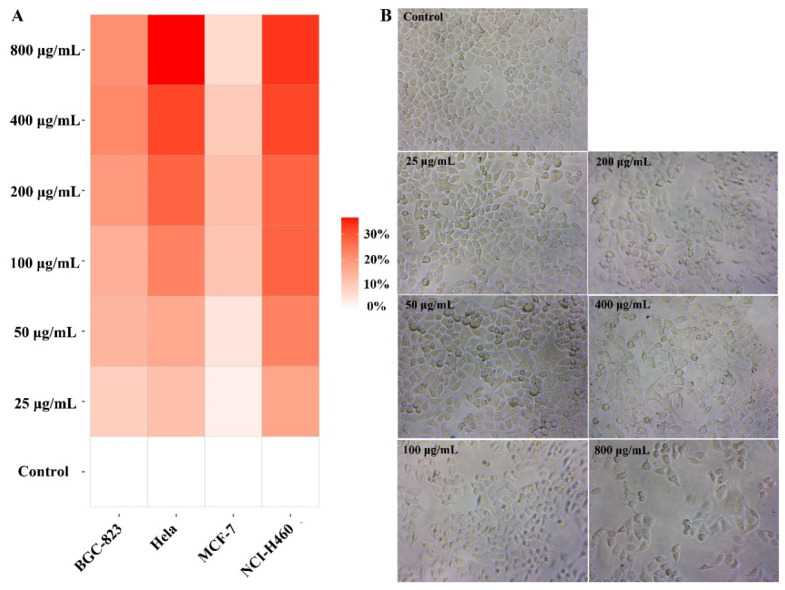
Effects of BBWPW on different cancer cells. BGC-823, HeLa, NCI-H460 and MCF-7 cancer cells were treated with different concentrations of BBWPW for 24 h, determined inhibition rate by MTT method (**A**) and observed the effect of BBWPW on HeLa cell morphology (**B**). *n* = 6.

We examined the cytotoxicity of BBWPW using normal fibroblasts (Vero cell) by MTT assay. We found that BBWPW has no toxicity as the concentration of BBWPW increases (Supplementary Materials Figure S4). In the same way, the MTT method was used to examine the inhibitory effect of BBWPW on the proliferation of HeLa, BGC-823, NCI-H460 and MCF-7 cells, and their inhibiting rates were shown in a heat map (Figure 3A). The darker the red, the better BBWPW inhibits the proliferation of cancer cells. Conversely, the lighter the red, the weaker the inhibition. We found that the inhibitory effects of BBWPW on HeLa and NCI-H460 cells was significantly stronger than on BGC-823 and MCF-7 cells, and increased in a concentration-dependent manner, reaching 33.75~36.57% at 800 μg/mL. In the following, we will mainly focus on the study of the inhibitory effects of BBWPW on Hela cells, as BBWPW has the strongest inhibitory effects on Hela cells.

The effects of BBWPW on the growth and morphology of HeLa cells were observed by the inverted light microscope. After the treatment of PBS or BBWPW for 24 h, we observed that control HeLa cells attached to the walls with irregular polygon and a distinct cytoskeleton. However, the cell morphology in the dose-treated group greatly changed. As the concentration of BBWPW increased, the cell adhesion rate decreased, the cell edges blurred, the number of adherent growth cells reduced, and a large number of globular suspended matter appeared (Figure 3B).

### 2.4. BBWPW Induces HeLa Cell Arrest in the G2/M Phase

MTT results showed that BBWPW inhibited the growth of Hela cells more than other cancer cells. Therefore, we investigated the anticancer mechanism of BBWPW based on the Hela cells.

**Figure 4:**
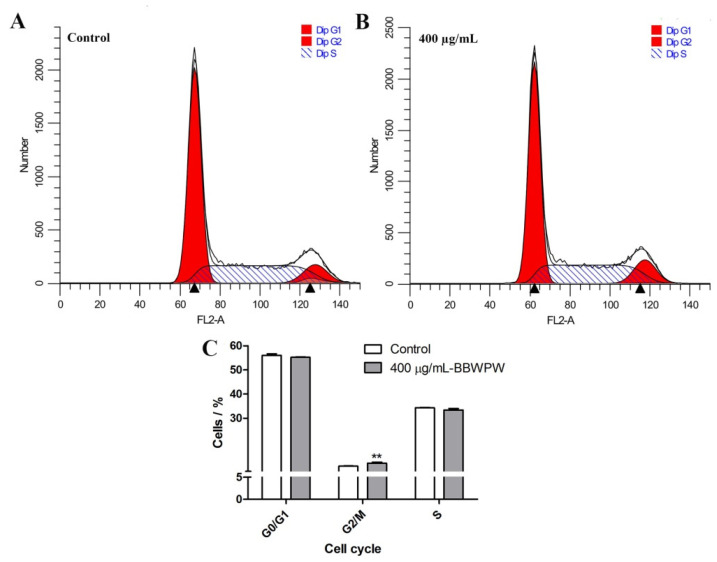
Cell cycle arrest of HeLa cells induced by BBWPW was detected. After being treated with PBS (**A**) or 400 μg/mL BBWPW (**B**) for 24 h, the cell-cycle phase of HeLa cells was examined by flow cytometry with PI staining. (**C**) The cell cycle arrest rate in different phases. *n* = 3, (**) represent *p* < 0.01 compared with the PBS group.

The effect of BBWPW on the cycle distribution of HeLa cells was demonstrated in Figure 4A,B. Compared with the untreated group, the proportion of cells in G0/G1 and S phases remained unchanged after BBWPW treatment for 24 h, but the proportion of cells in the G2/M phase increased from 10.13% to 11.37% (Figure 4C). These results indicated that G2/M phase cycle arrest might be an important reason for BBWPW inhibition of the proliferation of HeLa cells. In addition, the detection results of cell apoptosis showed that there was no significant change in the BBWPW-treated group compared with the untreated group (Supplementary Materials Figure S5).

**Figure 5:**
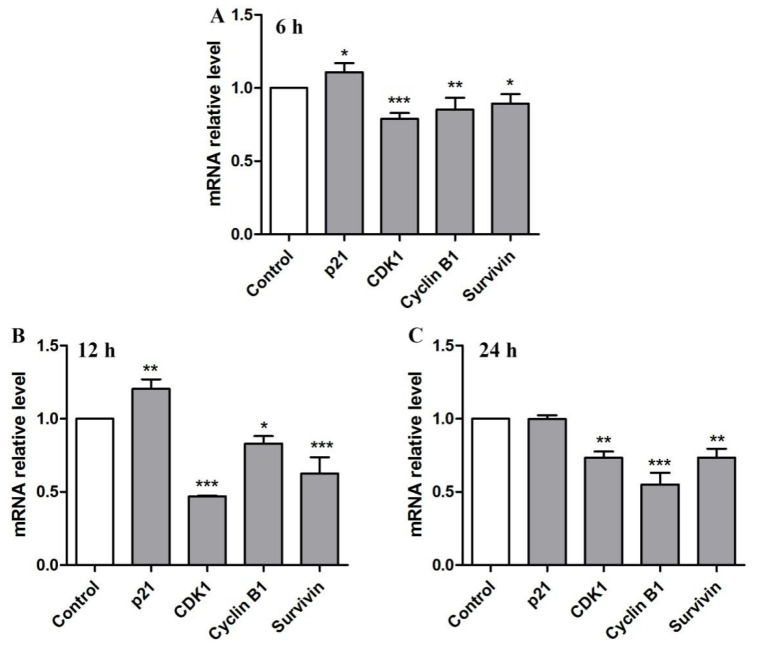
The effects of BBWPW on mRNA expression of cell cycle-related genes in Hela cells. The mRNA expression of genes was examined after treatment of 400 µg/mL BBWPW for 6 h (**A**), 12 h (**B**) and 24 h (**C**). PBS as the control group. *n* = 3. (***) *p* < 0.001, (**) *p* < 0.01 and (*) *p* < 0.05 compared with the control group.

To validate the cell cycle analysis results, we further examined four important G2/M-related cell cycle marker genes, including *p21*, *CDK1*, *cyclin B1,* and *Survivin*, by using RT-qPCR. It is well known that the formation of a complex by *CDK1* and *cyclinB1* is an important event of cell cycle arrest in the G2/M phase [14]. The Hela cells were treated with BBWPW for 6 h, 12 h, and 24 h, respectively. Compared with the control group, mRNA expression levels of *CDK1*, *cyclin B1*, and *Survivin* were significantly decreased (Figure 5). However, the expression of *p21* was significantly increased at 6 and 12 h, respectively.

### 2.5. Quantifying Analysis of RNA-Seq Data

**Figure 6:**
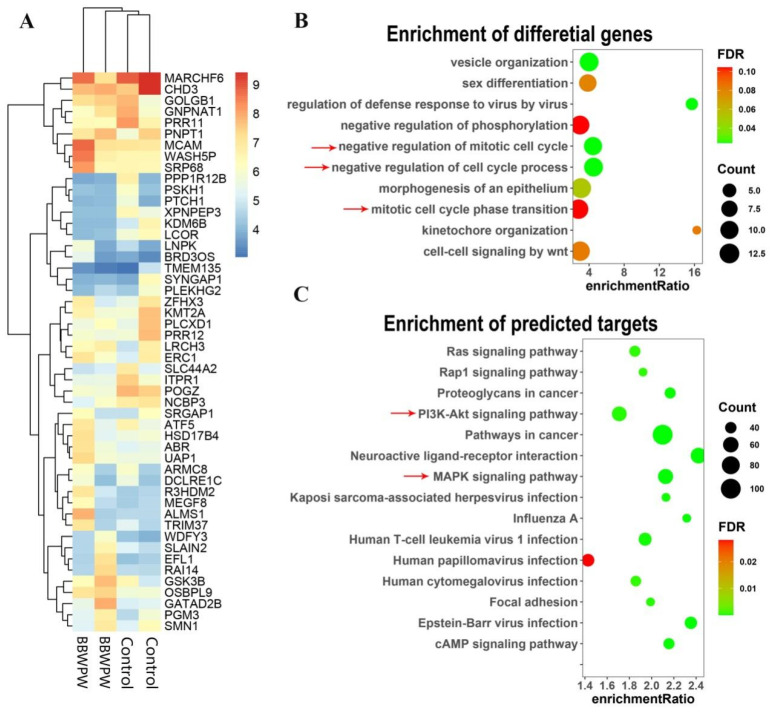
Analysis of RNA-seq data. (**A**) Hierarchical clustering of the samples by genes. In the heatmap, the 50 genes with the highest variance across samples were used. (**B**) Functional annotations of differential genes. Cell cycle pathways were indicated with red arrows. (**C**) Functional annotations of targets predicted by the transcriptome-based multi-scale network pharmacological platform. PI3k and MAPK pathways were indicated with red arrows. *n* = 2.

To detect the molecular mechanism of BBWPW for inhibiting cancer cells, a systematic transcriptomic analysis was performed in HeLa cells treated by BBWPW. The gene-level clustering analysis displayed that BBWPW-treated groups could be distinguished from the control groups (Figure 6A). The differential expression analysis in BBWPW-treated versus the control group found 160 significant differential genes (FDR ≤ 0.05; Supplementary Materials Table S1). Among them, 82 and 78 were upregulated and downregulated, receptively. Functional annotation analysis with WetGestalt [15] indicated that these differential genes were significantly enriched in the functions related to cell cycle, such as the GO biological processes “negative regulation of cell cycle process”, “mitotic cell cycle phase transition”, and “negative regulation of mitotic cell cycle”, consistent with the inhibiting effects of BBWPW on cell cycle in the flow cytometry analysis (Figure 6B).

Furthermore, to explore the molecular pathways targeted by BBWPW to inhibit the cancer cell cycle, we utilized the target prediction module of the transcriptome-based multi-scale network pharmacological platform (TMNP: http://www.bcxnfz.top/TMNP/, accessed on 15 January 2022) [16] to predict targets of BBWPW based on the BBWPW-induced transcriptional profiles (see details in Methods). TMNP predicted 985 potential targets for BBWPW (FDR ≤ 0.05; Supplementary Materials Table S2) and these targets were further characterized by over-representation analysis at the pathway level. The results displayed that many signal pathways were related to the cell cycle, such as “Pathways in cancer”, “MAPK signaling pathway”, “PI3K-Akt signaling pathway”, and “Ras signaling pathway” (Figure 6C; Supplementary Materials Table S3).

### 2.6. BBWPW Regulates the PI3K/Akt and MAPK Pathway

Previous research reported the PI3K/Akt pathway and MAPK pathway were important roles in regulating cell proliferation, differentiation, apoptosis and migration [17,18]. Therefore, according to transcriptomic analysis, *mTOR, AKT, BCL-2, BAD,* and *MKK3* genes related to these classic pathways were selected for experimental verification.

**Figure 7:**
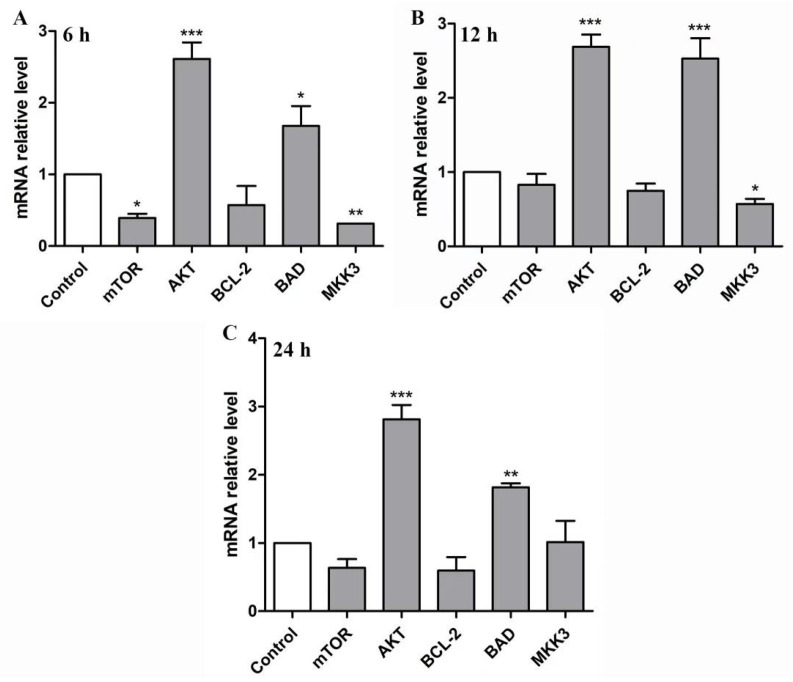
The mRNA expression of pathway-related genes in HeLa cells treated by BBWPW. The mRNA expression of genes was examined after treatment of 400 µg/mL BBWPW for 6 h (**A**), 12 h (**B**) and 24 h (**C**). PBS as the control group. *n* =3. (***) *p* < 0.001, (**) *p* < 0.01 and (*) *p* < 0.05 compared with the control group.

After being treated with 400 µg/mL BBWPW for 6 h, 12 h, and 24 h, the expression levels of these genes were measured by RT-qPCR. As shown in Figure 7, BBWPW treatment significantly up-regulated *AKT* and *BAD* expression at all-time points, down-regulated *mTOR* at 6 h, and down-regulated *MKK3* expression at 6 h and 12 h. In addition, the impact of BBWPW treatment on *BCL-2* is not significant. It can be seen that, except for *AKT* and *BAD*, the impact of BBWPW on other genes is relatively weak, and the expression of *mTOR* and *MKK3* was gradually restored within 24 h. Nevertheless, these results suggest that BBWPW could inhibit HeLa cell proliferation by regulating the expression levels of genes in the PI3K/AKT and MAPK pathway. Furthermore, it was found that the expression levels of *mTOR* and *MKK3* genes were correlated with the time of drug treatment. With the increase in drug action time, the *mTOR* and *MKK3* expression gradually recovered.

## 3. Materials and Methods

### 3.1. Experimental Materials and Chemical Reagent

Black bean was provided by the Soybean Genetics and Breeding Group of Shanxi Agricultural University. Standard monosaccharides, including glucose, mannose, galacturonic acid, rhamnose, fucose, arabinose, and galactose, were purchased from Solarbio (Beijing, China). The other chemicals, including lipopolysaccharide (LPS), 1-phenyl-3-methyl-5-pyrazolone (PMP), trifluoroacetic acid (TFA), 3-(4,5-Dimethylthiazol-2-yl)-2,5-diphenyltetrazolium bromide (MTT), and concanavalin A (ConA) were obtained from Sigma-Aldrich (Shanghai, China).

### 3.2. Cells

African Green Monkey kidney cell (Vero) was a kind gift from Prof. Yuanhao Qiu at Pingdingshan University. Different human cancer cell lines, including cervical cancer cell (HeLa), gastric cancer cell (BGC-823), large cell lung cancer cell (NCI-H460), and breast cancer cell line (MCF-7), were obtained from the scientific Research Platform of Tianjin Medical University. Vero, MCF-7, and HeLa cell lines were cultured in the DMEM medium and other cells were cultured with RPMI-1640 medium.

### 3.3. Extraction and Purification of Polysaccharides

The specific separation and purification process was shown in Supplementary Materials Figure S1. Tersely, the dried Black bean was decorticated, smashed, defatted, and then extracted 3 times using the distilled water at 80 °C for two hours. The supernatant was collected and precipitated with alcohol. The collected sediment was again dissolved by the distilled water to be dialyzed and lyophilized to get the Black bean crude extraction. Finally, a polysaccharide was separated from the crude extraction by using distilled water as the eluent through the DEAE-52 cellulose (Cl^−^ form) and Sephadex G-100 columns.

### 3.4. Determination Molecular Weight and Chemical Composition of Polysaccharide

The molecular weight of BBWPW was estimated by high-performance gel permeation chromatography (HPGPC). Three high-performance gel columns (Waters Ultrahydrogel 250, 1000 and 2000) with the same size (30 cm × 7.8 mm; 6 µm particles) were connected in series. The standard Dextrans (5.2, 23.8, 48.6, 148, 410 kDa) and BBWPW were eluted at 0.5 mL/min with 3 mM sodium acetate. The standard curve of log (Mw) vs. elution time (T) is: log (Mw) = −0.1719T + 11.58. In addition, the carbohydrate content of BBWPW was measured by the phenol–sulphuric acid method [19].

### 3.5. Monosaccharide Compositions

The polysaccharide BBWPW was hydrolyzed by 4 M TFA. The hydrolysis product of BBWPW and seven standard monosaccharides were derived with PMP and further examined by the high-performance liquid chromatographic (HPLC) [20]. Monosaccharide compositions of BBWPW were identified by comparing the retention times with standard monosaccharides and quantitatively determined by correction factor and peak area ratio.

### 3.6. Scanning Electron Microscopy and Infrared Spectroscopy Analysis

The morphology of the polysaccharide was observed by scanning electron microscopy (SEM). Lyophilized BBWPW powder was attached to the aluminum plate through double-sided conductive carbon tabs. After being sprayed with gold, the sample was observed by a scanning electron microscope (JSM-6490LV, JEOL, Akishima, Japan).

Infrared spectroscopy (IR) is used to detect information about functional groups and chemical bonds in BBWPW. In particular, the purified BBWPW was grounded with dried KBr and pressed into a pellet, which was then analyzed by the Fourier transform infrared spectrophotometer (TENSOR 27, BRUCK, Karlsruhe, Germany) between 400 and 4000 cm^−1^ [21].

### 3.7. Nuclear Magnetic Resonance Detection

Briefly, 50 mg BBWPW was dissolved into 2 mL D_2_O and lyophilized three times to completely substitute H with D. Then, the sample was dissolved into 0.5 mL D_2_O for NMR detection. The 1D and 2D spectra were recorded by using the Bruker AM 500 spectrometer (BRUCK, Karlsruhe, Germany).

### 3.8. Determination of Cytotoxicity and Anti-Cancer Activity

The logarithmic phase Vero cells were cultured in 96-well plates (1 × 10^5^/well), and then treated with various concentrations BBWPW (25, 50, 100, 200, 400, 800 μg/mL) for 24 h. Then, 10 μL MTT (5 mg/mL) was added and further incubated for 4 h at 37 °C. The supernatant was discarded. Hence, 100 μL dimethyl sulfoxide was added to dissolve Formazan. The optical density was measured at 570 nm to evaluate the cytotoxicity of BBWPW. HeLa, BGC-823, NCI-H460 and MCF-7 cells were used to determine the anti-cancer activity of BBWPW. Logarithmically growing cancer cells were digested by trypsin into a cell suspension and inoculated in 96-well plates (2 × 10^5^/mL). Then, as above, different cancer cells were treated with different concentrations of BBWPW. The morphological change was observed with an inverted light microscope (DMIL LED, Leica, Wetzlar, Germany) at the end of incubation. Finally, the supernatant was removed and DMSO was added to measure the absorbance value (A) at 492 nm with a microplate reader. The inhibition rate was calculated as follows:

$$\mathrm{Inhibition}\;\mathrm{rate}\;\left(\%\right)=\frac{A_{\mathrm{control}}-A_{\mathrm{sample}}}{A_{\mathrm{control}}}\times 100$$

### 3.9. Cell Cycle and Cell Apoptotic Detection

Cell cycle phase distribution and DNA content of BBWPW-treated cervical cancer cells were studied by flow cytometry [22]. In brief, logarithmic HeLa cells (5 × 10^6^/mL) were taken to a 6-well plate, supplemented with 400 μg/mL BBWPW, and incubated at 37 °C for 24 h. Then, cells were digested with trypsin, collected and washed twice with precooled PBS. The cells were fixed with 70% precooled ethanol overnight at 4 °C. Finally, PI staining was performed according to the kit instructions (Beyotime, code: C1052). The fixed cells were added with staining buffer, propidium iodide solution, and RNase, and then stained for 30 min in the dark room. The cell cycle change was detected by using the flow cytometry PE channel (Accuri^TM^C6, BD, Piscataway, NJ, USA).

An Annexin-FITC /PI apoptosis detection kit (Beyotime, code: CA1020) was used to detect the apoptosis of BBWPW-treated cervical cancer cells. Simply, HeLa cells were treated with 400 μg/mL BBWPW and incubated at 37 °C for 24 h. The cells were digested with trypsin and washed with precooled PBS. Then, the cells were re-suspended in 1 mL Binding Buffer at a concentration of 1 × 10^6^/ mL. 100 μL cell suspension was added to each labeled tube, and 5 μL Annexin V-FITC was added, dyeing at room temperature in the dark for 10 min. Thereafter, 5 μL PI was added, dyeing at room temperature in the dark for 5 min. After filtration, the samples were immediately detected by the flow cytometer (Accuri^TM^ C6 Plus, BD, Piscataway, NJ, USA). For steps in detail, refer to the kit instruction.

### 3.10. RNA Library Preparation, Sequencing and Data Processing

HeLa cells were treated with 400 μg/mL BBWPW for 12 h. Then the cells were digested with trypsin. Add the new medium for washing, centrifuge and discard the medium, and then wash and centrifuge with phosphate buffer. The RNA-seq analysis was performed by Majorbio Technology company (Beijing, China). Generally, total RNA was extracted using TRIzol reagent (TAKARA Biotechnology, Kyoto, Japan), and the pair-end sequencing was performed on Illumina HiSeq 4000 sequencer. The resultant FASTQ format data were cleaned by using fastp [23]. Sequence reads were aligned to the human reference genome to obtain the gene-level count datasets by the Salmon transcript quantification method [24]. Then, the tximport package [25] was used to aggregate the transcript-level quantification data to the gene-level estimated count matrices.

To perform the clustering analysis of the sequencing data, the RNA-seq count data were transformed into the homoskedastic data using the regularized-logarithm transformation (rlog) implemented in the R package DESeq2 (version: 1.28.1) [26]. The BBWPW-induced differential gene expression values versus the control group were calculated by DESeq2 on the raw counts.

### 3.11. Target Prediction Based on the Transcriptomic Data

We utilized the target prediction module of the transcriptome-based multi-scale network pharmacological platform (TMNP: http://www.bcxnfz.top/TMNP/, accessed on 15 January 2022) [16] to infer potential targets of BBWPW based on a BBWPW-induced transcriptional profile. The method can be used for samples belonging to two classes, e.g., BBWPW-treated vs. the control group in this work. The differential gene expression profile was ordered into a gene list based on the differential expression values between classes. Given the known gene modules induced by each target, the method can calculate similarity scores between the query gene list and the target-specific gene modules by using a method adjusted from that used in Gene Set Enrichment Analysis [27]. Normalized correlation scores (NCS) can be calculated between the query gene list and each target.

### 3.12. Real-Time Quantitative Polymerase Chain Reaction (RT-qPCR)

RT-qPCR was utilized to examine the expression of the pathway-related genes. In this work, HeLa cells were seeded in a 24-well plate. After treatment with 400 μg/mL BBWPW for 6 h, 12 h and 24 h, total RNA was extracted by using the Total RNA Kit I (OMEGA, Hartford, CT, USA) following the kit instructions. Then, total RNA was reversely transcribed into cDNA by using the PrimeScript^TM^ RT Master Mix (TaKaRa, code: RR36A). Quantitative real-time PCR was carried out using a TB Green Premix Ex Taq^TM^ II (TaKaRa, code: RR820A) by QuantStudio 6 (ABI, Carlsbad, CA, USA). The reaction conditions of RT-qPCR: predenaturation at 95 °C for 30 s, 40 cycles of denaturation at 95 °C for 5 s, and annealing at 60 °C for 30 s. The 2^−∆∆Ct^ method was utilized to determine the relative mRNA expression levels [28]. GAPDH was the internal control to normalize RT-qPCR data. The oligonucleotide sequence of the primers used by RT-qPCR was determined by searching the literature [29,30,31] and the Genebank database. These primers used in this study are shown in Table 2.

### 3.13. Statistical Analysis

Results were presented as mean ± SD. Significant differences were determined by one-way ANOVA or *t*-test with the GraphPad Prism 5.01.

## 4. Discussion

In the study, we identified a natural polysaccharide BBWPW from the black bean. Its average molecular weight was 2.4 × 10^4^ Da. NMR and monosaccharide composition results show that BBWPW is a glucan of α-(1→6)-Glc main chain. IR results showed that the conformation of the glucose is a pyran ring in BBWPW. BBWPW was extracted from the natural food black bean and should be nontoxic to normal cells. As we know, the saccharide compounds from natural plants have been found to have anticancer effects [32]. For example, polysaccharides from Ganoderma lucidum induce apoptosis of human colorectal cancer cells through MAPK/ERK activation [33], and polysaccharides from Fomes officinalis inhibits tumor proliferation [34]. Moreover, it has been reported that the protein hydrolysates of sprouted beans inhibit the growth of HeLa and C-33 cervical cancer cells [35]. Hence, we examined the anticancer activity of BBWPW. The results demonstrated that BBWPW inhibits the proliferation of four types of cancer cells, including HeLa, NCI-H460, BGC-823, and MCF-7 cells. Moreover, the inhibitory effects of BBWPW on HeLa and NCI-H460 cells were significantly stronger than on BGC-823 and MCF-7 cells. The different effects of BBWPW on these cell lines may be attributed to the difference of cell types or the mechanisms involved, as the four cancer cell lines have different tissue origins and pathogenesis. In the following, we will mainly focus on the study of the inhibitory effects of BBWPW on Hela cells, as BBWPW has the strongest inhibitory effects on Hela cells.

In general, cell proliferation inhibition can be carried out by inhibiting the cell cycle or promoting apoptosis [36]. Moreover, many natural products extracted from plants exert their anticancer activities by blocking the cell cycle and triggering cancer cell apoptosis [37]. For example, myricetin inhibited the proliferation of T24 bladder cancer cells by down-regulating cyclin B1 and cyclin-dependent kinase cdc2 to induce cell cycle arrest in the G2/M phase [38]. Here, the effects of BBWPW on apoptosis and cell cycle were detected. The results showed that BBWPW did not significantly promote HeLa cell apoptosis but induced G2/M the cell cycle arrest. Consistently, BBWPW significantly down-regulated mRNA expression levels of *CDK1, Cyclin B1*, and *Survivin* and up-regulated the expression of *p21*. It should be noted that, although the polysaccharide BBWPW significantly increases the level of p21 gene and decreases the levels of *Cyclin B1, CDK1,* and *Survivin,* the impact of BBWPW on these genes is still relatively weak. Correspondingly, in the flow cytometry test, BBWPW only induces the G2/M phase cycle arrest with a slight extent (from 10.13% to 11.37%) and the effects of BBWPW on the G0/G1 and S phases were not detected. Nevertheless, these results confirmed that the suppressive effects of BBWPW on the G2/M cell cycle result in proliferation inhibition.

To further detect the molecular path of BBWPW to induce G2/M cell cycle arrest, a transcriptome-based target inference approach was utilized to predict the potential biological targets of BBWPB based on the gene expression profiles. It was found that BBWPW could significantly regulate genes in the MAPK and PI3K/Akt pathways. In fact, many reports have showed that the two pathways were deeply involved in cancer cell proliferation. Therefore, targeting the MAPK and PI3K/Akt pathways would be an effective therapeutic option to control the proliferation and migration of cancer cells [39]. The results showed that BBWPW treatment mainly regulated *AKT* and *BAD* expression, and only slightly impact *mTOR* and *MKK3* expression. It has been known that the activation of the PI3K/Akt pathway can suppress the activity of *BAD* gene. As a regulator of *BCL-2* family members, *BAD* protein positively regulates cell apoptosis by forming heterodimers with BCL proteins [40]. Many studies have shown that glucans exhibit anticancer and antiproliferative effects by targeting the growth factors or toll-like receptors, which can be related to the PI3K/Akt pathway [41,42]. Therefore, we speculated that BBWPW might inhibit the proliferation of cancer cells through regulating the PI3K/Akt pathway by targeting the growth factors and toll-like receptors.

In addition, many studies have reported that polysaccharides usually display obvious immune-stimulating effects. For example, Yan Ma et al. confirmed that soy oligosaccharides (SBOS) promote splenic lymphocyte proliferation, which can be used as potential prebiotics to improve immune function [43]. The polysaccharide from Codium fragile induced anti-cancer immunity by activating natural killer cells [44]. Astragalus polysaccharides inhibit cancer cell proliferation by direct killing effect and improving immune function [45]. It has been known that the harmony of the human immune system is the main reason for preventing cancer [46]. Thus, the best strategy to prevent and fight cancer is to improve the body’s immunity [47]. In the future, we will examine the immunoregulatory capacity of BBWPW and study the potential cooperative effects of its immune and anticancer functions.

## 5. Conclusions

In conclusion, our study obtained a novel α-1,6-glucan from black beans and found that the glucan could inhibit the proliferation of cancer cells via G2/M cell cycle arrest. The molecular mechanism was detected by combining bioinformatic and experimental methods and displayed that the suppressive effects of the glucan on the G2/M cell cycle can be attributed to the PI3K/Akt and MAPK pathways. Furthermore, we expect to confirm the anti-cancer activity of BBWPW in animal models and even clinical studies. These studies will accelerate the development and utilization of black bean polysaccharides as potential functional food ingredients or anticancer drugs.

## Figures and Tables

**Table 1:** The C/H chemical shifts of BBWPW.

Sugar Residues	C1 H1	C2 H2	C3 H3	C4 H4	C5 H5	C6 H6a	H6b
α-1,6-Glc	97.77 4.91	71.41 3.52	73.40 3.65	69.57 3.45	70.22 3.85	65.60 3.93	3.69

**Table 2:** Primers for RT-qPCR.

Genes	Primer Sequences (5′−3′)
*GAPDH*	F: CATGAGAAGTATGACAACAGCCT; R: AGTCCTTCCACGATACCAAAGT
*p21*	F: GCGGAACAAGGAGTCAGACA; R: GAACCAGGACACATGGGGAG
*CDK1*	F: TTGAAACTGCTCGCACTTGG; R: TCCCGGCTTATTATTCCGCG
*Cyclin B1*	F: CTGCTGGGTGTAGGTCCTTG; R: TGCCATGTTGATCTTCGCCT
*Survivin*	F: GGCCCAGTGTTTCTTCTGCT; R: ATGAGGGTGGAAAGCAACCC
*mTOR*	F: TCCGAGAGATGAGTCAAGAGGAGTC; R: GCTGGAAACCAATTCAAAAATGTG
*BAD*	F: CGGAGGATGAGTGACGAGTTTGT; R: ATCCCACCAGGACTGGAAGACTC
*AKT*	F: ACCTTCCATGTGGAGACTCCTGAG; R: GTCCATCTCCTCCTCCTCCTGC
*BCL-2*	F: CATGTGTGTGGAGAGCGTCAAC; R: CTTCAGAGACAGCCAGGAGAAATC
*MKK3*	F: CGCGGATCCAAAGAAGCAGACC; R: CGGAATTCAAAGCCGGGATAGAGG

## Data Availability

Not applicable.

## Supplementary Materials

The following supporting information can be downloaded at: https://www.mdpi.com/article/10.3390/molecules28041971/s1, Figure S1: Extraction and purification scheme of the polysaccharide BBWPW from Black bean; Figure S2: High-performance gel permeation chromatogram of BBWP; Figure S3: Electron microscopy images and Infrared spectroscopy of BBWPW; Figure S4: The cytotoxicity of BBWPW to Vero cells; Figure S5: Apoptosis of HeLa cells induced by BBWPW; Table S1: 160 significant differential genes (FDR ≤ 0.05) in BBWPW-treated versus untreated cases; Table S2: Potential targets predicted by the target module in TMNP; Table S3: Over-representation analysis of 985 potential targets for BBWPW (FDR ≤ 0.05) at the pathway level with WetGestalt.

## Sample Availability

Samples of the compound BBWPW are available from the authors.
