# Supplementary material for: A Natural Glucan from Black Bean Inhibits Cancer Cell Proliferation via PI3K-Akt and MAPK Pathway
Source: Molecules. 2023 Feb 19;28(4):1971. doi: 10.3390/molecules28041971 (PMC9961350; doi:10.3390/molecules28041971)
Supplement: Supplementary file 1 [file molecules-28-01971-s001.zip › Supplementary Figures.pdf]

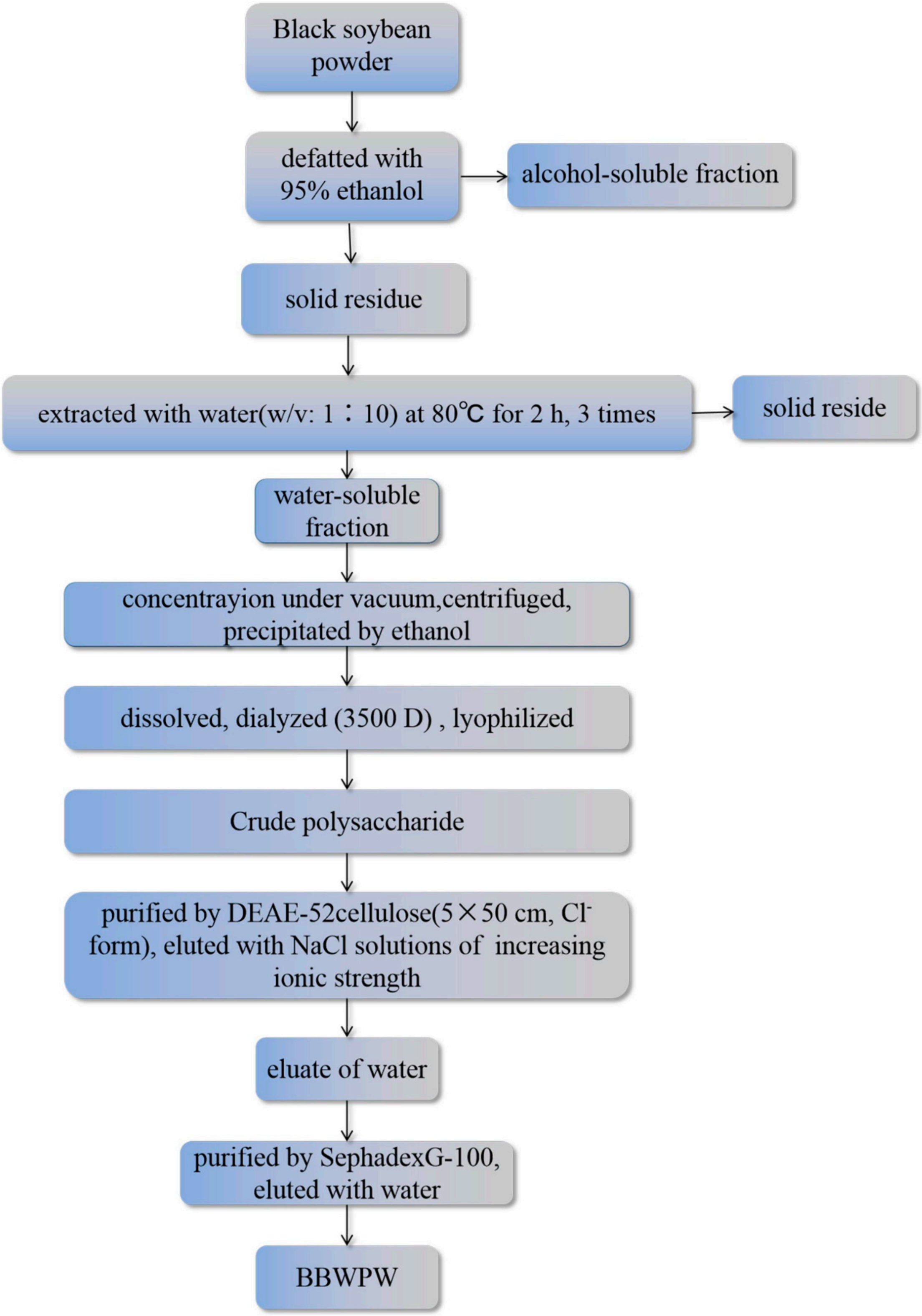

Figure S1. Extraction and purification scheme of the polysaccharide BBWPW from Black bean

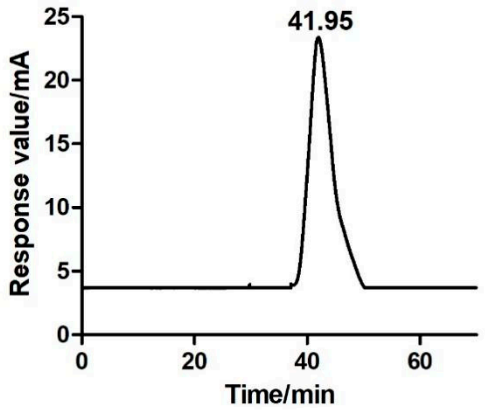

Figure S2. High-performance gel permeation chromatogram of BBWPW

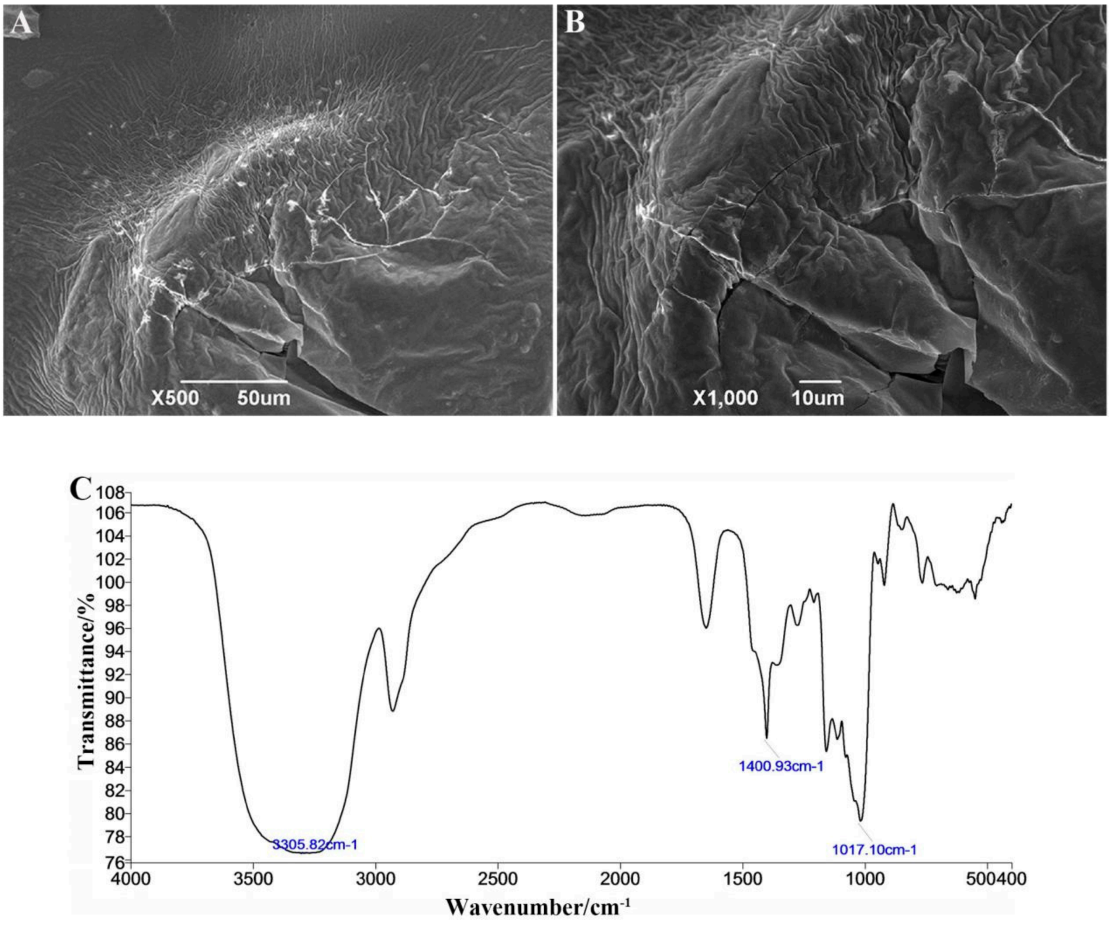

Figure S3. Electron microscopy images (A, B) and Infrared spectroscopy (C) of BBWPW.

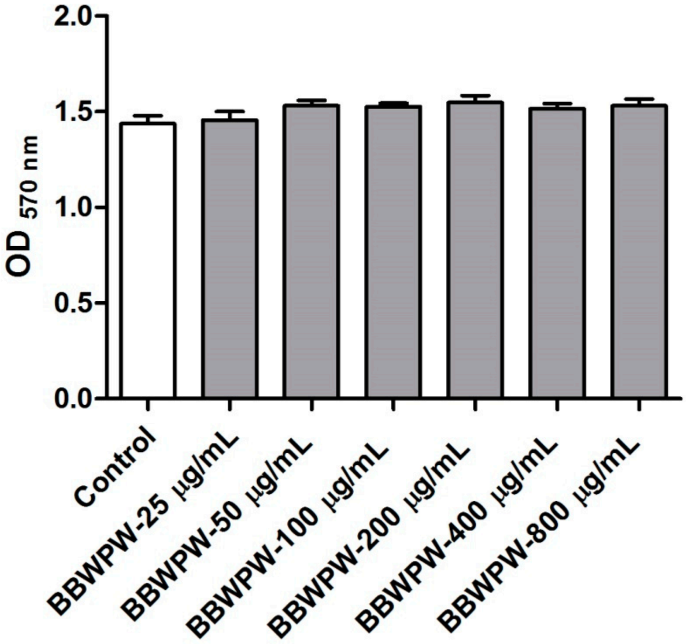

Figure S4. The cytotoxicity of BBWPW to Vero cells.

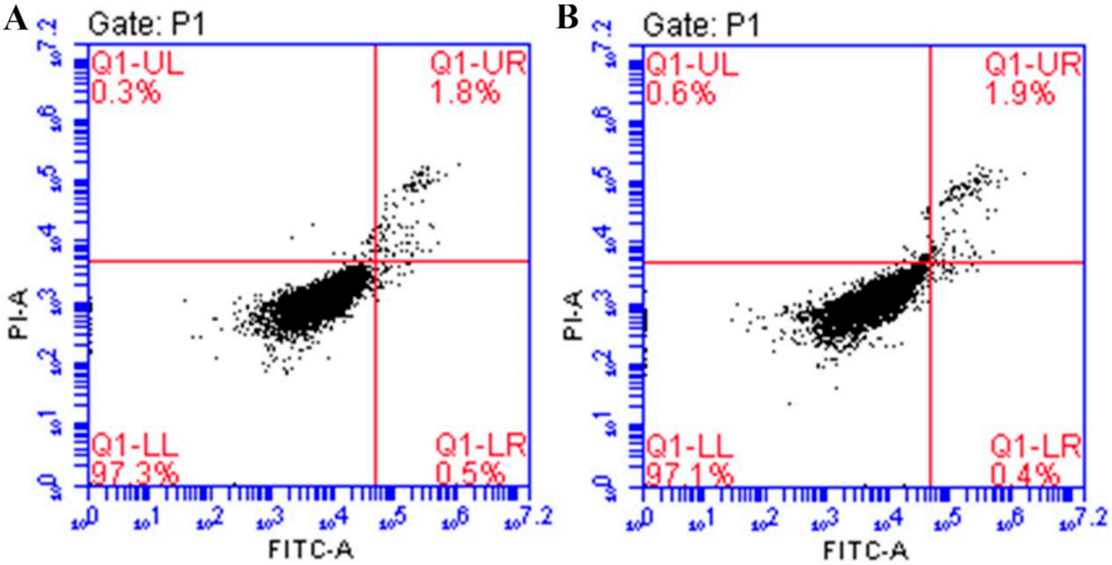

Figure S5. Apoptosis of HeLa cells induced by BBWPW. After treated with PBS (A) or 400 μg/mL BBWPW (B) for 24 h, the apoptosis of HeLa cells were assessed by flow cytometry analysis after staining with Annexin V-PI .
